# Editorial: Urinary tract infections: molecular mechanisms of pathogenesis

**DOI:** 10.3389/fcimb.2023.1191478

**Published:** 2023-04-12

**Authors:** Naofumi Naga, Wieslaw Kaca

**Affiliations:** ^1^ Department of Applied Chemistry, College of Engineering, Tokyo, Japan; ^2^ Department of Microbiology, Institute of Biology, Jan Kochanowski University, Kielce, Poland

**Keywords:** urinary tract infection (UTI), pathogeneses, ureolytic activity, biofilm, tissue injury

For this Research Topic, the pathogeneses connected to Urinary Tract Infections (UTI) have been investigated from different points of view. Duran Ramirez et al. focused on catheter-associated urinary tract infections. They presented high-throughput detection of urease activity with a semi-quantitative assay for testing potent (P. mirabilis) or weak (S. aureus) urease producers. Ballén et al. compared clinical isolates of Klebsiella pneumoniae strains for antibiotic resistance patterns and virulence factors. They reported that the isolates from urine with strong biofilm-forming abilities were more resistant to the antibiotics than those from the respiratory tract or blood. The authors concluded that the acquisition of mobile genetic elements could promote not only the spread of antimicrobial resistance genes but also virulence genes that evolve into virulent pathotypes of K. pneumoniae. Wu et al. presented the molecular mechanisms of bladder tissue injury caused by an overreaction of the immune response, which was induced by uropathogenic E. coli. They proposed the up-regulation of the alpha-mannose receptor by C5a/C5aR1, which facilitates UPEC adhesion via FimH lectin on type 1 fimbriae. Gmiter and Kaca reviewed the fate of P. mirabilis strains on solid surfaces, focusing on adhesion, biofilm formation, and the swarming phenomenon.

We thank all the authors contributing to this Research Topic despite the coronavirus pandemic. We hope that the presented studies will encourage future discoveries about the pathogenesis of UTIs.

## Author contributions

NN: Conceptualization, Supervision, Writing-review and editing, WK: Conceptualization, Supervision, Writing-original draft preparation, Project administration. All authors contributed to the article and approved the submitted version.

